# Supplementary material for: Plasma Dephosphorylated-Uncarboxylated Matrix Gla-Protein in Systemic Sclerosis Patients: Biomarker Potential for Vascular Calcification and Inflammation
Source: Diagnostics (Basel). 2023 Nov 24;13(23):3526. doi: 10.3390/diagnostics13233526 (PMC10706314; doi:10.3390/diagnostics13233526)
Supplement: Supplementary file 1 [file diagnostics-13-03526-s001.zip › diagnostics-2686282-supplementary.pdf]

diagnostics

MDPI

Table S1. Clinical characteristics of the validation cohort of SSc patients (*n* = 29).

Clinical Features	SSc Cohort *n* = 29
**Age at inclusion, mean ± SD, y**	60.3 (13.3)
**Female sex**	22 (75.9)
**Disease duration, median, IQR, y**	1.0 (6.9)
**Skin involvement**	
Limited	21 (72.4)
Diffuse	5 (17.2)
Sine sclerosis	3 (10.3)
**Antibody**	
Centromere	18 (62.1)
Topoisomerase I	6 (20.7)
RNA polymerase III	1 (3.4)
Other (Ku, Pm-ScL)	2 (6.9)
ANA only	2 (6.9)
**Digital ulcers**	11 (37.9)
**ILD**	11 (37.9)
**Pulmonary hypertension**	2 (6.9)
**Immunosuppressive therapy**	0
**Creatinine, median, IQR, umol/L**	77 (26)
**CRP median, IQR, mg/L**	2.0 (3.0)
**sIL2r, median, IQR, U/mL**	453 (417)
**dp-ucMGP, median, IQR, pmol/L**	589 (370)
**CVD at baseline**	5 (17.2)

Data are presented as numbers (%) unless otherwise indicated. IQR: interquartile range; ILD: interstitial lung disease; ANA: anti-nuclear antibodies; CRP: c-reactive protein. sIL2r: soluble interleukin 2 receptor. Dp-ucMGP: Dephosphorylated and uncarboxylated Matrix Gla Protein. ¥ Defined as the date of the first non-Raynaud's phenomenon symptom. Reference ranges for creatinine are 60–115 umol/L, CRP < 10 mg/L, and sIL2r < 600 U/mL.
